# Determination of zearalenone in raw milk from different provinces of Ecuador

**DOI:** 10.14202/vetworld.2021.2048-2054

**Published:** 2021-08-09

**Authors:** Byron Puga-Torres, Miguel Cáceres-Chicó, Denisse Alarcón-Vásconez, Carlos Gómez

**Affiliations:** 1 Laboratorio de Control de Calidad de Leches, Facultad de Medicina Veterinaria y Zootecnia, Universidad Central del Ecuador, Quito, Ecuador; 2 Doctorado en Ciencia Animal, Facultad de Zootecnia y Escuela de Postgrado, Universidad Nacional Agraria La Molina, Lima-Perú

**Keywords:** Ecuador, enzyme-linked immunosorbent assay, raw milk, zearalenone

## Abstract

**Background and Aim:**

Zearalenone (ZEA) is a mycotoxin from the fungus Fusarium. ZEA can adopt a similar configuration to 17b-estradiol and other natural estrogens. Problems in the reproductive function of humans and animals have been reported for ZEA and its metabolites. This study aimed to determine ZEA in raw milk produced in representative milk production areas in Ecuador.

**Materials and Methods:**

A total of 209 samples were obtained in April and November 2019 (rainy season) and June and August 201ue wa9 (dry season). A competitive enzyme-linked immunosorbent assay techniqs used to detect ZEA concentrations.

**Results:**

ZEA was determined in 99.5% (208 of 209) of the samples; however, all samples were below the maximum limits allowed (0.03-1 mg/L) in food for direct human consumption according to the Food and Agriculture Organization and European legislations. The mean (range) concentration was 0.0015 (0-0.0102) mg/L. The results did not vary significantly (p≥0.05) by cantons, provinces, weather, climate regions, types of producers, and production systems according to Wilcoxon and Kruskal–Wallis non-parametric tests. There were significant differences only between the months under study (p≤0.05).

**Conclusion:**

ZEA in raw milk from Ecuador does not represent a threat to public health. However, it is recommended to continue analyzing ZEA due to its presence in milk. It could also be present with other mycotoxins that cause harmful synergistic and additive effects to consumers.

## Introduction

Milk production in 2018 reached 864,101 million liters/year worldwide, where 81.4% was from cattle, with South America ranking fourth place in milk production, accounting for 9.2% of the world’s production. Ecuador ranks fifth in the region [1], reaching 6.65 million liters/day, with a per capita consumption between 90 and 95 kg/person/year [2].

Zearalenone (ZEA) is a mycotoxin from the fungus *Fusarium* (fusariotoxins), similar to deoxynivalenol and fumonisins [3]. It affects various types of food worldwide, such as wheat, corn, barley, sorghum, sesame seed, hay, silage, and animal feed [4,5]. ZEA is a lactone resorcyl acid [6] mainly produced by *Fusarium graminearum*, *Fusarium culmorum*, and, on a lesser extent, *Fusarium equiseti*, *Fusarium gibbosum*, *Fusarium oxysporum*, and *Fusarium moniliforme*. Its maximum presence occurs at temperatures between 20°C and 25°C. If the water activity is higher, there is greater ZEA production [7], so rains can significantly increase ZEA concentrations in wheat. ZEA remains at very low concentrations in the absence of moisture even when severely affected by *Fusarium* [8].

ZEA can adopt a similar configuration to 17β-estradiol and other natural estrogens [4]. ZEA undergoes biotransformation by reducing the ketone group of carbon 7 by a hydroxyl group, obtaining α-zearalenol (α-ZEA) [9] that is 3-4 times greater than the original compound [10] and β-zearalenol (β-ZEA). There can also be a reduction in the double bond between carbons 11 and 12, forming zearalanone (ZAN) [11]. The affinity and relative power of estrogenicity in descending order are α-ZEA, α-ZEA (α-ZAN), β-ZEA, ZAN, ZEA, and β-ZEA (β-ZAN) [12]. Problems in the reproductive function of humans and animals have been reported for ZEA and its metabolites [13]. The maximum permitted levels of ZEA in cow’s milk and its derivatives have not been determined despite its high production and consumption worldwide because its elimination in milk is very low [14] due to the molecular weight and lipophilic level of these metabolites [10]. The transformation of ZEA to its metabolites is done in the liver (primarily) and by rumen protozoa [15]. It can then be transferred to raw milk in the forms of ZEA and α-ZEA [16]. In dairy cows’ feed, with three different levels of corn contaminated with ZEA, the conversion rates were 0-0.0075%, not representing a risk due to insignificant transition rates [17]. The microbiota of the rumen, formed by protozoa and bacteria, of healthy bovines constitutes the first line of defense, decreasing mycotoxin contamination of food. In sick animals or when there is excess contamination by mycotoxins in their diets and/or, in turn, dietary changes, minimizing the risk of excretion in milk may be ineffective [10]. There could be alterations in the degradation of this mycotoxin, including the proportion of contaminated concentrated feeds, ruminal fermentation, or associated liver alterations in bile formation and volume [18].

As it is impossible to guarantee a food completely free of mycotoxins [19], different regulations worldwide have established limits between 0.03 and 1 mg/L ZEA in food in general [20-24]. However, in Ecuador, no maximum limits have been established for ZEA within the Ecuadorian Technical Standards (NTE-INEN). There are also no studies of this mycotoxin in raw milk. Therefore, this study aimed to determine its presence in major milk-producing and representative provinces of Ecuador, taking into consideration, its two climatic regions (Andean and coastal regions), times of the year (dry and rainy), types of producer (small, medium, and big), and production systems (extensive, intensive, and mixed).

## Materials and Methods

### Ethical approval

This study did not need contact with animals. So, this study did not require ethical approval.

### Study period and area

The study was conducted from April to November 2019. Due to its natural resources, Ecuador has different milk production systems [25]. The province of Pichincha in North Central Ecuador, one of the most representative regions, represents ~16% of the country’s total milk production and has a temperature between 5°C and 20°C, 70-75% humidity, and 2500-3200 m above sea level (asl). Manabí follows with ~12% and has a temperature between 20°C and 35°C, 82% and 90% humidity, and 150-300 m asl. Santo Domingo de los Tsáchilas represents ~4% of Ecuadorian production and has a temperature between 21°C and 32°C, 80-90% humidity, and 150-300 m asl [2,26-28].

The rainy period is between November and May and the dry period is between June and September [29]. ZEA was sampled in these provinces, as they share similar climatic conditions and characteristics (climate, topography, soils, pastures, etc.) to the rest of the milk-producing provinces of Ecuador [26]. Together, they represent more than 30% of the total production of Ecuadorian milk [2].

### Sample collection

A total of 209 samples were collected in April and November 2019 (rainy season) and June and August 2019 (dry season), where 72.3% of the samples were from Pichincha (151 of 209), 22.9% from Manabí (48 of 209), and 4.8% from Santo Domingo de los Tsáchilas (10 of 209). Regarding climatic season, 53.1% were collected during the rainy season (111 of 209) and 46.9% during the dry season (98 of 209). In April, June, and August 2019, 49 samples (23.5%) were taken each month, whereas the remaining 29.7% (62 of 209) of the samples were taken in November 2019.

Milk samples were collected in an approximate quantity of 100 mL from bulk tanks on the farms following the provisions of the NTE-INEN-ISO 707: “Milk and dairy products. Guidelines for sampling” [30]. The samples were then transferred to the Milk Quality Control Laboratory of the Faculty of Veterinary Medicine and Zootechnics of the Central University of Ecuador. During transport, a cooler with refrigerants was used to maintain the temperature between 2°C and 5°C and the samples were then stored at −20°C until the respective analysis.

A survey was carried out among the farmers to stratify them according to (1) province: Pichincha, Manabí, and Santo Domingo de los Tsáchilas; (2) climate region: Andean (cold) or coastal (heat); (3) type of producer: Small, medium, and big (1-20 cows in production, 21-100 animals, and >100 cows, respectively); and (4) production systems: Extensive (grazing animals), intensive (stagnant cows), and mixed (grazing and stagnant animals). Independent of the production system, most animals were fed with fresh and concentrated grass and sometimes with silo and hay.

### ZEA analysis in milk by enzyme-linked immunosorbent assay (ELISA)

All milk samples during each collection month were thawed at room temperature (10-20^o^C) and skimmed by centrifugation for 10 min at 4000×*g*. After centrifugation, the upper fat layer was removed, and skim milk was analyzed following the recommended procedure by the test kit MaxSignal^®^ ZEA (ZON) ELISA (Bioo Scientific Corporation, Austin, TX, USA) with a microplate reader Stat Fax 3200-2260 (Awareness Technology, Inc., Palm City, FL, USA) to 450 nm.

The method was based on a competitive colorimetric ELISA test, where ZEA was covered in the plate wells. After the addition of the substrate, the intensity of the resulting color was inversely proportional to its concentration in the milk. The kit has high cross-reactivity with ZEA (138%), α-ZEA (91%), and α-ZAN (69%). The limit of detection was as low as 0.000015 mg/L, and the limit of quantification was 0.004 mg/L, with high sensitivity (0.0001 mg/L) and high reproducibility. It has a specificity (cross-reactivity) of 100% (ZEA), 138% (ZAN), 91% (α-ZEA), 21% (β-ZEA), 69% (α-ZAN), and 6% (β-ZAN). It also includes standard solutions of 0, 0.1, 0.25, 0.5, 1.5, 4.5, and 0.025 mg/L used to generate regression curves between ZEA concentration and optical density.

For the test, a 20 μL skim milk sample was diluted with 35% methanol (Merck, Darmstadt, Germany), with a dilution factor of 1:10. All reagents were kept at 10-20^o^C until use and homogenized by slow inversion. The procedure used a 1X wash solution. For the ZEA antibody mix, 1 volume of the ZEA antibody was mixed with 1 volume of the antibody conjugate (horseradish peroxidase [HRP] #2). This mixture was used within 5 min after its preparation, as a longer storage time can result in lower detection values.

All analyses were performed in duplicate to achieve a sufficient number of samples, calculate the standard deviation and coefficient of variation, and validate the data. Initially, 50 μL of each ZEA standard were added, depending on the kit, in different wells in ascending order, that is, from low concentration to high concentration. In the following wells, 50 μL of each sample were added followed by 100 μL ZEA-HRP conjugate to all wells using a multichannel pipette. The well solutions were homogenized manually by gentle plate movements for 1 min. After incubating the plate at 10-20^o^C for 30 min, the solution was completely decanted from the wells, and the liquid was desiccated. The plate was washed three times with 300 μL of 1×wash solution. After the last wash, the plate was inverted and gently tapped on paper towels. A 100 μL TMB substrate was added to each well. The plate was gently mixed for 1 min and incubated at 10-20^o^C for 15 min, covering the plate with adhesive. A 100 μL solution was finally placed as a stop buffer to stop the enzyme reaction. The bottom of the plate was wiped with a cloth to avoid moisture or fingerprints that might interfere with the readings and was immediately read on the plate reader with a 450 nm wavelength.

To design the standard curve based on the average relative absorbance (%), the following formula can be used, derived from each reference standard based on its concentration (ng/mL) on a logarithmic curve: Relative absorbance (%)=(standard absorbance or sample×100/zero standard absorbance). With the mean relative absorbance values for each sample, the concentration of each mycotoxin was determined from the standard curve. However, to obtain the calibration curve, the ELISA analysis program MaxSignal® in Excel was used.

### Statistical analysis

All results were expressed as the mean, minimum, and maximum concentrations of ZEA. The Shapiro–Wilk test (p<0.05) was performed to confirm the normality of the data, resulting in an extremely low p=2.2e^−16^ (p≤0.05); therefore, non-parametric tests were used [31]. The differences in ZEA concentrations were analyzed in terms of cantons, provinces, types of producers, production systems, and sampling months by the Kruskal–Wallis test, whereas the time and climatic region were analyzed by the Wilcoxon test. When there were significant differences in the Kruskal–Wallis test, a *post hoc* analysis was performed using the Mann–Whitney U-test on each pair of groups using a Bonferroni correction to decrease the probability of incurring error type I. The free statistical software RStudio version 1.2.5019 (RStudio, Inc., Boston, MA, USA) was used with a level of statistical significance of p<0.05.

## Results

At the end of the investigation, 99.5% (208 of 209) of the raw milk samples analyzed were positive for ZEA, but all milk samples analyzed were below the maximum limits allowed by different food regulations, ranging between 0.03 and 1 mg/L [20-24]. Only one sample was under the detection limit (UDL).

**Figure-1:**
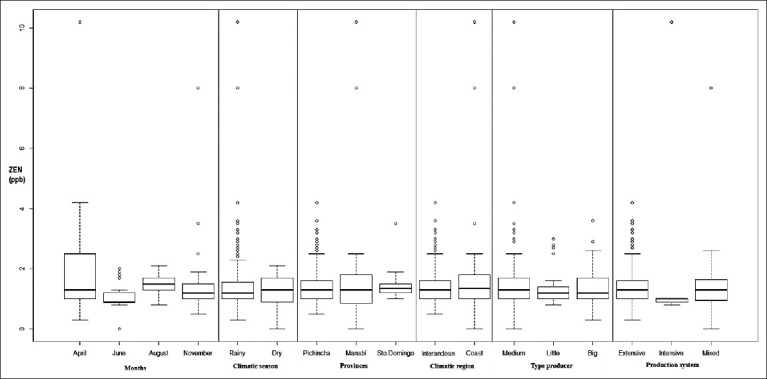
Boxplot with values according to months, climatic seasons, provinces, climatic regions, types of producer, and production systems.

Tables-1 and 2 and Figure-1 show the results obtained by cantons, provinces, months, climatic regions, climatic seasons, types of producer, and production systems. By cantons, the mean was 0.0015 mg/L and the maximum was 0.0102 mg/L (Table-1), both from Chone Canton of Manabí. By provinces, where the maximum value was 0.0102 mg/L, where two were from Manabí. The lowest mean (0.0014 mg/L) was from Pichincha, whereas the highest mean (0.0016 mg/L) was from Manabí and Santo Domingo de los Tsáchilas (Table-1). By cantons and provinces, the p-value was 0.5683 and 0.6541, respectively (p≥0.05), without significant differences between the cantons and provinces. By climatic region, the maximum value was 0.0102 mg/L, both from the coastal region. The lowest mean (0.0014 mg/L) corresponded to the sierra region, whereas the largest (0.0016 mg/L) was from the coastal region (Table-1). The p-value was 0.8426 (p≥0.05), without significant differences between the climatic regions.

**Table 1:** Minimum, mean, and maximum values of ZEA obtained by cantons, provinces, and climatic regions (n=209).

Variable		Minimum (mg/L)	Mean (mg/L)	Maximum (mg/L)	SD (mg/L)
Canton					
Mejía	Pichincha	0.0008	0.0015	0.0021	0.0010
Quito	Pichincha	0.0008	0.0016	0.0036	0.0011
Cayambe	Pichincha	0.0005	0.0015	0.0032	0.0010
Pedro Moncayo	Pichincha	0.0009	0.0018	0.0042	0.0011
Rumiñahui	Pichincha	0.0009	0.0014	0.0027	0.0011
Chone	Manabí	UDL	0.0020	0.0102	0.0011
El Carmen	Manabí	0.0003	0.0014	0.0025	0.0010
Alluriquín	Santo Domingo de los Tsáchilas	0.0010	0.0016	0.0035	0.0007
Province					
Pichincha		0.0005	0.0014	0.0042	0.0009
Manabí		UDL	0.0016	0.0102	0.0011
Santo Domingo de los Tsáchilas		0.0010	0.0016	0.0035	0.0007
Climatic region					
Coastal		UDL	0.0016	0.0102	0.0011
Inter-Andean		0.0005	0.0014	0.0042	0.0009

SD=Standard deviation, UDL=Under the detection limit

**Table 2:** Minimum, mean, and maximum values of ZEA obtained by months, climatic seasons, types of producer, and production systems (n=209).

Variable	Minimum (mg/L)	Mean (mg/L)	Maximum (mg/L)	SD (mg/L)
Month				
April	0.0003	0.0019	0.0102	0.0011
June	UDL	0.0011	0.0020	0.0004
August	0.0008	0.0015	0.0021	0.0009
November	0.0005	0.0014	0.0080	0.0009
Climatic season				
Rainy	0.0003	0.0016	0.0102	0.0010
Dry	UDL	0.0014	0.0080	0.0011
Type of producer				
Small	0.0008	0.0014	0.0030	0.0010
Medium	UDL	0.0015	0.0102	0.0010
Big	0.0003	0.0014	0.0036	0.0010
Production system				
Extensive	0.0003	0.0014	0.0040	0.0010
Intensive	0.0008	0.0028	0.0102	0.0011
Mixed	UDL	0.0015	0.0080	0.0010

SD=Standard deviation, UDL=Under the detection limit

Based on the results per month (Table-2), the UDL value corresponded to June, whereas the maximum (0.0102 mg/L) was in April. The lowest mean (0.0011 mg/L) was found in June, and the highest mean (0.0019 mg/L) was found in April. The p-value was 2.253e^−07^ (p≤0.05), with significant differences between April and June, between June and August, between June and November, and between August and November.

By climatic season, the UDL value corresponded to the dry season, and the maximum (0.0102 mg/L) corresponded to the rainy season (Table-2). The lowest mean (0.0014 mg/L) corresponded to the dry season, and the highest mean (0.0016 mg/L) corresponded to the rainy season. The p-value obtained was 0.4469 (p≥0.05), without significant differences between the climatic seasons.

By types of producer (Table-2), the UDL value and the maximum (0.0102 mg/L) were from the medium producers. The lowest mean (0.0014 mg/L) was from the large producers, whereas the highest mean (0.0015 mg/L) was from medium producers. The p-value was 0.8225 (p≥0.05), without significant differences between the types of producers.

By production systems (Table-2), the UDL value corresponded to the mixed type, whereas the maximum value (0.0102 mg/L) corresponded to the intensive type. The lowest mean (0.0014 mg/L) corresponded to the extensive type, and the highest (0.0028 mg/L) corresponded to the intensive type. The p-value was 0.4368 (p≥0.05), without significant differences between the production systems.

## Discussion

There are no reports of ZEA determination in raw milk from Ecuador, so this research is the pioneer. The mycotoxin was found in 99.5% (208 of 209) of the raw milk samples, with a mean of 0.0015 mg/L. The results showed much higher values than those found in various studies. For example, in China, the presence of ZEA was determined only in 23.3% of the raw milk samples, 16.7% in pasteurized milk, and 25% in milk powder of several dairy farms and different supermarkets in Beijing [32]. In Monte Carlo milk, ZEA was determined at a mean of 0.00039 mg/L and was associated with other mycotoxins. ZEA was also detected in 9% of 185 cow’s milk-based infant formulas [33], with corn as the main source [34]. In Argentina, ZEA was determined at the concentration of 0.0013 mg/L in raw milk, where corn silage and balanced feed are the largest mycotoxin reservoirs [35]. In Portugal, ZEA was found in 100% of the milk samples from a dairy farm [36]. In all cases, these values were below the mean of this study.

In Turkish breast milk, ZEA was also determined at a mean of 0.0002 mg/L [37]. In infant formulas of four different brands from Italy, ZEA was determined in 9% (7 of 185) of the samples, with a maximum value of 0.0008 mg/L; a-ZEA was determined in 26% (9 of 185), with a maximum value of 0.0129 mg/L; and β-ZEA was determined in 28% (53 of 185), with a maximum value of 0.0732 mg/L. No a-ZAN or β-ZAN was found [33].

More studies are needed to assess the importance of milk and derivatives as a source of estrogen for humans [38]. Although ZEA is not directly carcinogenic [10], it has disruptive effects on hormonal balance due to its similarity to natural estrogens [39,40] and may cause reproductive system diseases, including prostate, ovary, cervix, and breast cancers [41]. ZEA is also toxic to liver cells [42], ZEA can also affect the development of gamete and embryogenesis in humans and animals [43] and lead to precocious puberty in girls, fertility disorders and reproduction in women [44], testosterone reduction, spermatogenesis, and even feminization in men [45], causing damage to germ cells and testicular structure [43].

ZEA can bioaccumulate in the body [41]. On its own, ZEA can cause oxidative stress in the small intestine, ileum, and mesenteric lymph nodes [46]. However, when combined with several mycotoxins, ZEA causes major problems in intestinal function [47] and cytotoxicity in human Caco-2 cells [48]. In milk, ZEA is frequently found together with ochratoxin A or aflatoxin M1 (AFM1) [49]. This is important because, in a parallel study, AFM1 was found in the same raw milk in 100% of the samples, with a mean of 0.0774 μg/kg [50].

Therefore, it is necessary to take appropriate measures to reduce risks to human and animal health [51,52]. Because mycotoxins present in raw milk are very stable and heat treatments, such as pasteurization, cannot remove them [53], it is only useful with high temperatures between 237°C and 306°C (but the organoleptic characteristics of milk are lost). Different techniques to remove mycotoxins in food are classified into (1) physical (microwave, extrusion, heating, ultraviolet light, gamma radiation, and adsorption agents, such as bentonite and aluminosilicates), (2) chemical (oxidants, such as ozone and hydrogen peroxide, and hydrolases, such as aldehydes, acids, and bases), and (3) biological (enzymes of microorganisms, lactic acid bacteria, yeasts, etc.) [54]. One of the widely used techniques is the addition of an antimycotoxin additive to the diet of dairy cows, which can prevent many harmful effects on animals [55].

Modern methods include obtaining cell-free supernatants from whey after fermentation by kefir granules (CIDCA AGK1) that act on the growth of *F. graminearum* and the consequent production of ZEN [56] and the biocatalyst from *Gliocladium roseum* (ZENG) with a high degradation performance toward ZEN and its toxic derivatives a-ZEA and a-ZAN [57]. *Lactobacillus kefiri* (KFLM), *Kazachstania servazzii* (KFGY7), and *Acetobacter syzygii* strains are also used. When consumed together with kefir, they reduce the gastrointestinal absorption of mycotoxins [58].

## Conclusion

This investigation is the first to detect ZEA in raw milk from Ecuador. All raw milk samples analyzed had ZEA levels (with a mean of 0.0015 mg/L) below those allowed by national and international regulatory organizations. Therefore, its presence does not constitute a threat to public health. However, much attention should be paid, and more research is needed, as 99.5% of the samples had levels of this mycotoxin. ZEA may be present with other mycotoxins in milk that cause harmful synergistic and additive effects to consumers.

## Authors’ Contributions

BP and CG: Designed the study, wrote the manuscript, and participated in conducting the experiment. BP, MC, and DA: Collected the samples. BP, MC, and DA: Processed and analyzed the data. All authors read and approved the final manuscript.
